# Linking the impact of aspiration to host variables using the BOLUS framework: support from a rapid review

**DOI:** 10.3389/fresc.2024.1412635

**Published:** 2024-07-12

**Authors:** Phyllis M. Palmer, Aaron H. Padilla

**Affiliations:** ^1^ Department of Speech and Hearing Sciences, University of New Mexico, Albuquerque, NM, United States; ^2^ Department of Rehabilitation Services, Presbyterian Healthcare Services, Albuquerque, NM, United States

**Keywords:** dysphagia, aspiration, BOLUS framework, aspiration pneumonia, oral health, swallow impairment, swallow disorder

## Abstract

**Purpose:**

The purpose of this rapid review was to identify the level of evidence for a previously proposed theoretical framework to assess risks associated with prandial aspiration using the host as a central theme.

**Methods:**

Covidence software was used to search two databases (PubMed and Web of Science). PEDro scale was utilized to determine the quality of individual studies. Data points were evaluated for level of support and determined to be either *conclusive*, *suggestive*, *unclear*, or *not supportive*. Within each component of the framework, data points were clustered to determine the level of evidence as *strong*, *moderate*, *insufficient*, or *negative*.

**Results:**

The rapid review process resulted in a limited number of publications investigating host variables impact on outcomes for patients with swallowing disorders. Overall, it yielded 937 articles, of which, upon review, 16 articles were selected for data extraction. There was a strong level of evidence to support that (a) as viscosity and density of aspirate increased, so did the likelihood of general medical complications, (b) poor oral care and oral health increase the risk of a pulmonary or general medical complication, and (c) the presence of oropharyngeal or laryngeal tubes increases the risk of a pulmonary consequence. There was moderate evidence to support the impact of amount and frequency of aspiration on outcomes. There was insufficient evidence to determine relationships for all other aspects of the BOLUS framework.

**Conclusion:**

Additional evidence to support the BOLUS framework was obtained; however, the number of studies was limited. A more thorough review such as a systematic review should be employed.

## 1 Introduction

When choosing a treatment, one must balance the cost of benefit and associated risk. This often involves choosing between various risks with a goal of achieving the most favorable long-term outcome given the status of the host and the medical prognosis. Management of swallowing disorders requires this same balance. Prandial aspiration comes with risk, and that risk may be mitigated or enhanced depending on host variables and treatment choices. The literature clearly supports that the presence of aspiration alone does not guarantee a negative adverse event. In a landmark study evaluating predictors of aspiration pneumonia, Langmore et al. (1) states that while aspiration is required, it will not result in an adverse event unless “*the material aspirated is pathogenic to the lungs and if the host resistance to the inoculum is compromised*” (p. 76). This notion is further supported in more recent literature [e.g., (2–4)]. In essence, the risk of an adverse event associated with prandial aspiration is not solely, or even strongly, dependent on the presence of the aspiration event (1, 4). Yet, when aspiration is observed, clinicians may opt to terminate (at least temporarily) oral intake and shift patients to alternative nutrition pathways. When considering discontinuing oral alimentation (i.e., non-oral feeding or NPO) because of prandial aspiration, clinicians must balance the cost of reduced oropharyngeal muscle engagement due to limited oral intake with the impact potential aspiration will have on lung health and patient morbidity. Given that the evidence in the literature supports the fact that risks associated with aspiration are multifaceted, we need to visualize ways to implement the available evidence in patient care.

**Figure 1:**
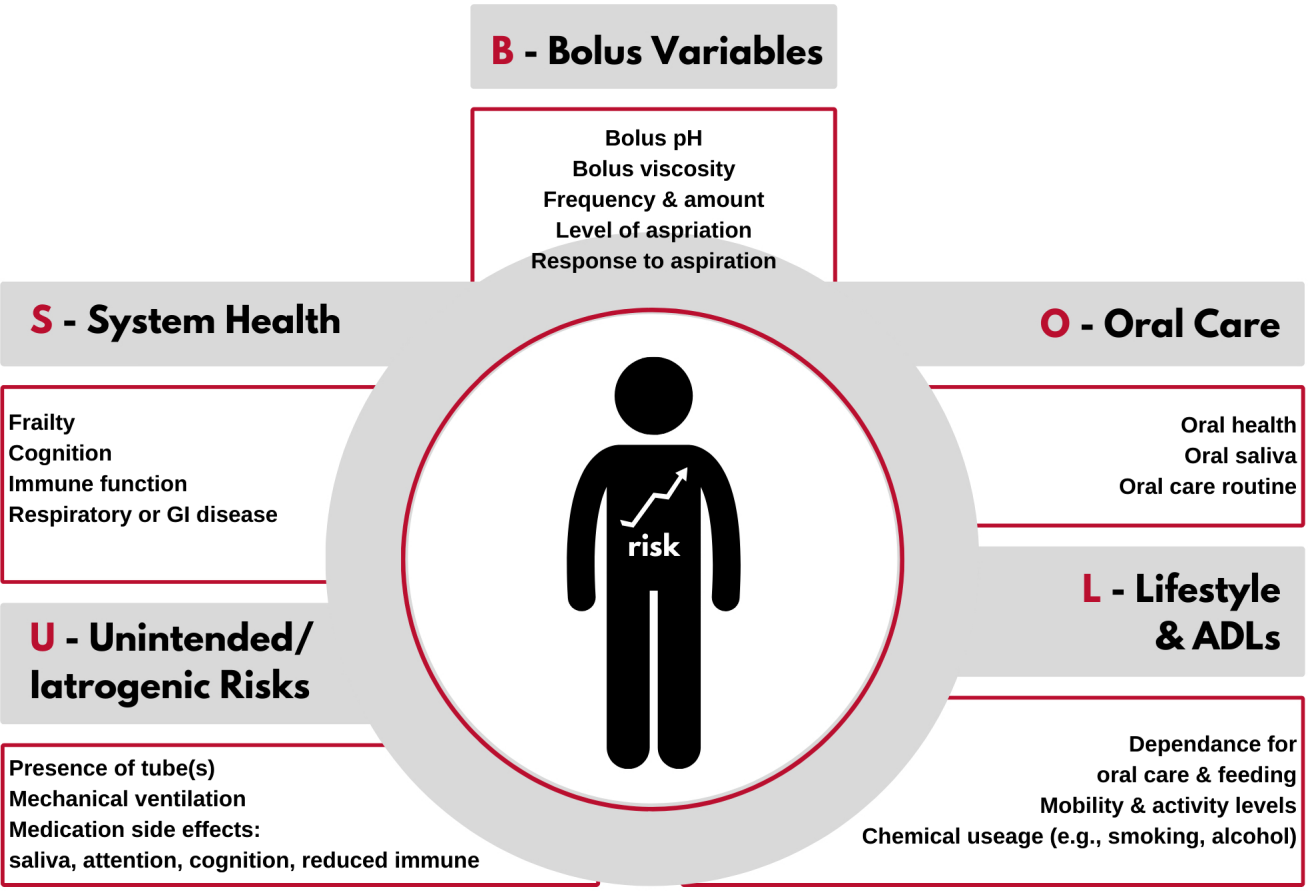
A schematic of the variables incorporated in the BOLUS framework.

To demystify the linkage between aspiration and risk of an adverse event, armed by current evidence, Palmer and Padilla (2) outlined a theoretical framework designed to aid dysphagia clinicians in assessing the cost of prandial aspiration for individuals with a swallowing disorder. Using the acronym BOLUS, the framework includes set categories each associated with a list of clinical questions to elucidate potential risks that may influence the likelihood of an adverse event from aspiration. Figure 1 displays a schematic of the BOLUS framework. Each letter in BOLUS represents a category of the framework. Specifically, B represents variables associated with the aspirated bolus, such as density, pH, and frequency of aspiration; O represents oral health and oral care; L represents lifestyle choices such as level of activity or smoking status, as well as performance of activities of daily living; U represents unintended or iatrogenic risks, such as the presence of a tracheotomy tube or ventilator, and S represents general system health and other co-morbidities. Table 1 lists the questions associated with each category. (See Appendix A in Supplemental Materials for links to YouTube videos on the BOLUS framework). Simplistically, “yes” responses to the various questions within the framework are presumed to indicate an increase in the risk of an adverse event. Once the questions have been answered, the clinician considers which of the identified risks are modifiable. For example, in the case of poor oral care routines, a modifiable risk, training may be provided to improve current oral care. However, that is not the case with an individual who has well-managed respiratory disease, which is not a modifiable risk factor.

**Table 1:** Questions associated with the BOLUS framework.

B	• Does aspiration of thick or dense material increase the risk of an adverse event? • Does frequent or large amounts of aspiration increase the risk of an adverse event? • Does aspiration of acidic material increase the risk of an adverse event?
O	• Does poor oral health or oral care increase the risk of an adverse event? • Does poor saliva production increase the risk of an adverse event?^a^
L	• Does level of mobility or physical activity alter the risk of an adverse event? • Does smoking or alcohol consumption alter the risk of an adverse event? • Does dependance for activities of daily living (e.g., oral care and feeding) alter the risk of an adverse event?^a^
U	• Does presence of a tube in the oropharynx (or airway) alter the risk of an adverse event? • Does use of mechanical ventilation alter the risk of an adverse event?^a^ • Does use of medications with side effects that impact saliva production, attention, level of alertness, or cognition alter the risk of an adverse event?^a^
S	• Does poor general health alter the risk of an adverse event?^a^ • Does presence of respiratory or GI disease alter the risk of an adverse event?^a^ • Does level of cognition alter the risk of an adverse event?^a^ • Does frailty alter the risk of an adverse event? • Does reduced immune function alter the risk of an adverse event?^a^ • Does absence of a cough response or a weak cough alter the risk of an adverse event?^a^

All questions assume that aspiration is present. Adverse events of interest in this manuscript are listed in Table 3.

^a^ Indicates that no data were identified in this rapid review related to this question.

This framework serves to move beyond mere considerations of the presence of aspiration and places the host as a central focus to guide clinical decisions. By considering the questions proposed in the BOLUS framework and identifying which risk factors are modifiable, clinicians can make evidence-based clinical recommendations while embracing the level of risk of an adverse event. We seek to identify the cost-benefit comparison of recommending that an individual continue with oral alimentation (given the mealtime recommendations of the clinicians) vs. the risk of having an individual be NPO for a period of time. By better understanding the associated risk(s) of oral intake vs. total alternative nutrition, we may better support healthy outcomes for our patients with prandial aspiration.

The aim of this project was to identify the level of support in the literature for a link between various components (noted in this manuscript as the BOLUS framework) and various adverse events (noted in this manuscript as outcome measures). We assessed the clinical usefulness of the proposed BOLUS framework by employing a rapid review of the literature. A rapid review prioritizes efficiency without compromising rigor. Although a systematic review is the preferred method, we opted to start with a rapid review for several reasons. Primarily, despite a more than 20-year history of published literature that documents variables associated with adverse events from prandial aspiration, there have been limited efforts to translate these findings into clinical practice. Furthermore, there lacks a clear framework connecting this evidence into a structured and consistent clinical approach. The observation that the implementation of empirical evidence into clinical practice is protracted, with a reported average latency of 17 years (5), underscores the necessity for expeditious methodologies. Consequently, a rapid review was chosen as an initial step to accelerate the transition from theoretical framework to clinical implication, should the framework demonstrate potential as a clinical tool.

## 2 Methods

### 2.1 Search strategy

PubMed and Web of Science electronic databases were searched to identify relevant peer-reviewed articles published between the years 1998 and 2021 using search terms indicated in Table 2. Specifically, we searched for studies that addressed swallowing disorders with aspiration along with at least one aspect of the BOLUS framework (i.e., bolus variables, oral health and oral care, lifestyle choices, unintended risk factors, and system health). Identified studies were imported into Covidence (6), a software platform designed to assist with structured literature reviews. Using a defined criterion, two levels of review were conducted including (a) abstract and title review, and (b) full text review before finalizing articles to be included in the data extraction process. At each level of review, two reviewers independently performed the required tasks. Articles were eliminated if they (a) did not address the presence of aspiration and an associated outcome from aspiration, or (b) did not contain novel data. Outcome measures of interest are listed in Table 3. At any point in the decision tree, discrepancies across two reviewers were resolved by consensus.

**Table 2:** Search terms (i.e., medical subject hearing, or MeSH) and limits employed during the search.

Search terms (MESH terms)	(respiratory tract infection* AND aspiration* OR dysphagia) AND (bolus* OR oral* OR activity OR mobil* OR tube* OR oxygen* OR cannula OR respiratory* OR GI* OR immune function)
Limits	Language: English only Years: 1998–2021

**Table 3:** List of outcome measures.

• Pulmonary ⚬ Aspiration pneumonia/aspiration pneumonitis ⚬ Respiratory disease exacerbation [e.g., asthma or chronic obstructive pulmonary disease (COPD)] ⚬ Lung complications/damage
• General medical complications ⚬ New onset infection/fever [e.g., urinary tract infections (UTI)] ⚬ Dehydration • Survival

To assess the completeness of the identified literature, we compared the citations from Palmer and Padilla (2), which laid the groundwork for the BOLUS framework, to the articles identified in the rapid review. This was done simply by noting the percentage of the citations from the Palmer and Padilla tutorial that were also identified using the rapid review procedure.

### 2.2 Assessment of study quality

Once studies were identified for inclusion, an assessment of study quality was conducted using the PEDro scale (7), which is a tool designed for evaluating quality of clinical studies. The total number of “yes” responses on this 11-item yes/no scale served as a measure of the strength of the study design. This scale was completed independently by two raters. When discrepancies occurred between the two raters, raters reviewed the study and discussed the PEDro scores until discrepancies were resolved.

### 2.3 Assessment of BOLUS framework

Articles selected for inclusion were divided by the framework categories. For example, articles that addressed impact of bolus thickness are included under the B framework category. Studies were included in the analysis of all BOLUS framework categories in which their data applied. For example, if a study evaluated the impact of bolus thickness and mobility, it would be included under the B and L framework categories. Study data were then organized by outcome measures (Table 3). Inspired by Perry et al. (8), each extracted data point was evaluated for level of support and determined to be either *conclusive*, *suggestive*, *unclear*, or *not supportive* (Table 4)*.* This determination was based on three parameters— (1) statistical findings for the specific outcome measure, (2) the direct applicability of the evidence, and (3) the PEDro score given to the study from which the data were extracted. For a data point to be assigned as *conclusive* support, (a) an outcome measure was statistically validated with either a significant *p*-value or large effect size with respect to one of the BOLUS framework components, (b) data were obtained from human subjects with known aspiration, and (c) the data was extracted from a study with a PEDro score of 4 or higher. For *suggestive* support, the criteria for conclusive were not met and at least one of the following was true: (a) outcome measures were *near* significance or had at least a moderate effect size, or (b) outcome measures were significant, but either data were obtained from an animal model, the presence of aspiration was not clearly or adequality determined, or the outcome measure was not directly assessed (e.g., the study measured level of hydration instead of presence of dehydration). In addition, data were extracted from a study with a PEDro score of 2 or higher. *Unclear* support was determined when (a) statistics were missing or weak, or the information provided in the study was difficult to interpret with a clear relationship to the BOLUS framework, and/or (b) the data was extracted from a study with a PEDro score of 0 or 1. A data point was determined to be *not supportive* if any of the following were true: (a) significant *p*-values or large effect size that went against the relationship between any component in the BOLUS framework and any outcome measure, or (b) non-significant *p*-values that tested the hypothesis of the relationship between any component in the BOLUS framework and one of the outcome measures. In addition, data were extracted from a study within a PEDro score of 2 or higher. Ratings of level of support for each extracted data point (i.e., given outcome measure for a specific aspect of the BOLUS framework) were completed independently by two raters and compared for consistency. When disagreement occurred, these were discussed until consensus was achieved.

**Table 4:** Criteria employed to assign level of support for individual datapoints extracted from each article and overall strength of evidence for BOLUS framework categories.

Scale for evaluation of level of support from individual data points	
Conclusive	• Statistically significant outcome measure (e.g., either *p* < 0.1 or effect size of 0.8 or greater) • Data obtained from human participants with known aspiration • Data were extracted from a study with a PEDro score of 4 or higher
Suggestive	• One of the following is true ○ Outcome measure was *near* significance or had at least a moderate effect size ○ Outcome measure was significant AND data were not obtained from human participants OR the study question did not directly address the outcome measure • Data were extracted from a study with a PEDro score of 2 or higher
Unclear	• One of the following is true ○ Statistics were missing or weak ○ Information was difficult to interpret with a clear relationship to the BOLUS framework ○ Data were extracted from a study with a PEDro score of 0 or 1
Not supportive	• One of the following is true: ○ Significant *p*-values or large effect size that goes against the relationship between any component in the BOLUS framework and any outcome measure ○ Non-significant *p*-values that tested the hypothesis of the relationship between any component in the BOLUS framework and one of the outcome measures ○ A PEDro score of 2 or higher
Scale to assess strength of evidence for BOLUS framework	
Strong	• One of the following is true: ○ At least two *conclusive* supports ○ One *conclusive* support and two *suggestive* supports • When strong evidence was determined based on the above definition and there was also at least one data point that was rated as *not supportive*, the rating of strong evidence was reduced to moderate evidence.
Moderate	• One of the following is true: ○ At least one *conclusiv*e support and one *suggestive* support ○ Three *suggestive* supports • When moderate evidence was determined based on the above definition and there was also at least one study that was rated as *not supportive*, the rating of moderate evidence was reduced to insufficient evidence
Insufficient	• One of the following is true: ○ None of the criteria for strong or moderate evidence were met ○ Less than three data points.
Negative	• Any number of *not supportive* ratings with or without *unclear* ratings, and no *conclusive* or *suggestiv*e ratings

**Figure 2:**
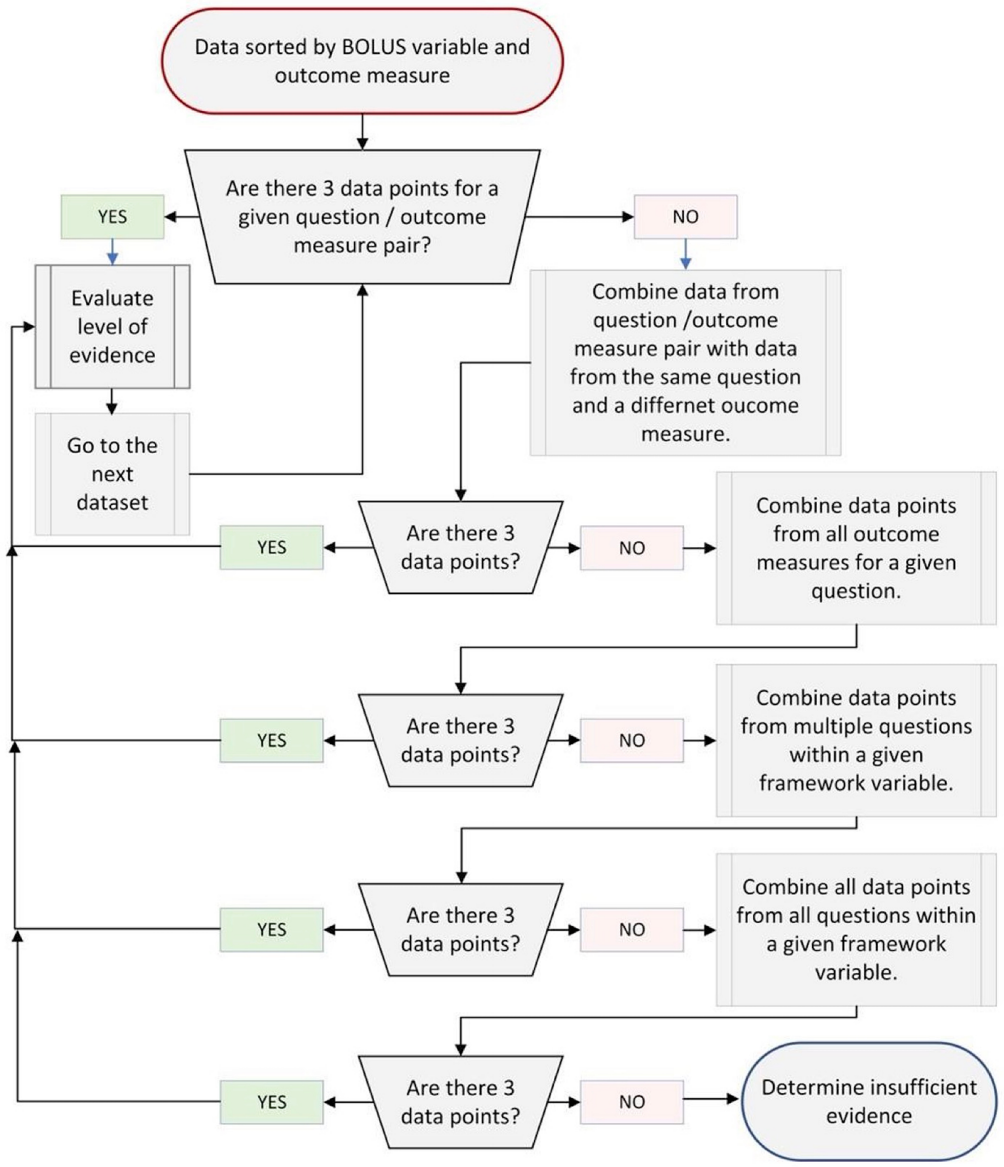
Flowchart to determine datapoints to be included in level of evidence analysis.

Once support ratings (i.e., conclusive, suggestive, unclear or not supportive) were completed for each extracted data point from each article, these were sorted first by BOLUS framework component (Figure 1) and then by framework question (Table 1). For each question, data were clustered by outcome measure to determine overall level of evidence for a given question and the impact on a given outcome measure. The overall level of evidence for each question and outcome measure pair in the BOLUS framework was determined when at least three datapoints were available for evaluation. To optimize the evidence evaluation, when there were less than three data points per question-outcome measure pair, data points were clustered using the following rules (Figure 2). Data across multiple outcome measures within a given question in the BOLUS framework were combined and the evidence was assessed across outcome measures. When this procedure did not yield a minimum of three data points, data points were further collapsed across several questions within a BOLUS framework component.

The cumulative evidence for each clustered subset of the BOLUS framework was determined using the following criteria (Table 4). The evidence was determined to be strong for a given cluster in the framework if there were (a) at least two *conclusive* supports (as defined above) or (b) one *conclusive* support and two *suggestive* supports. When strong evidence was determined based on the above definition and there was also at least one data point that was rated as *not supportive*, the rating of strong evidence was reduced to moderate evidence. Moderate evidence for a given cluster in the framework was determined if there was (a) at least one *conclusiv*e support and one *suggestive* support, or (b) three *suggestive* supports. When moderate evidence was determined based on the above definition and there was also at least one datapoint that was rated as *not supportive*, the rating of moderate evidence was reduced to insufficient evidence. Insufficient evidence was determined when none of the above criteria were met or there were insufficient data points (i.e., less than three data points) to be eligible to meet the above criteria. In other words, if there were less than three data points across all questions within a given component, the data were deemed as insufficient evidence to judge the usefulness of that BOLUS framework component. Negative evidence was determined when only *non-supportive* ratings were extracted or not supportive rating were combined with *unclear* ratings. In other words, when no *supportive* or *conclusive* support was identified for any data point in the cluster, and there is at least one *not supportive* rating within the cluster, then the cluster was rated as having negative evidence.

## 3 Results

### 3.1 Article selection

The rapid review search yielded 937 articles (Supplementary Figure S3 in Supplemental Materials). Of those, 18 duplicates were removed leaving 919 articles. Title and abstract screening resulted in elimination of 634 articles; full text screening eliminated an additional 269 articles. A total of 16 articles met the criteria for extraction.

The rapid review identified 3 of the 101 articles cited in Palmer and Padilla (2) yielding 3% of articles that might be important in the review but were not identified using the search term and methods defined. In addition, the rapid review added 13 articles that were not included in the Palmer and Padilla tutorial. [See Appendix B in Supplemental Materials for a comprehensive list of references comparing the Palmer and Padilla (2) tutorial with the results of the rapid review].

### 3.2 Bolus framework evidence

Across the 16 studies, 31 data points were extracted for inclusion in this investigation — B = 12 datapoints, O = 13 datapoints, L = 3 datapoints, U = 3 datapoints, and S = 0 datapoints. Table 5 summarizes the individual datapoints and the resulting level of evidence.

**Table 5:** An overview of data support for aspects of the BOLUS framework organized by the impact of a specific variable on the risk of an adverse event.

Bolus variables (12 data points extracted from six of the 16 articles.)						
	Does aspiration of thick and dense material increase risk of …			Does aspiration of frequent and large amounts increase risk of …		
	Pulmonary	Survival	Other	Pulmonary	Survival	Other
conclusive	0	0	3	0	1	0
suggestive	2	1	1	2	0	0
unclear	1	0	0	0	0	0
not supportive	0	1	0	0	0	0
Overall evidence	Insufficient		Strong	Moderate		
Oral health/Oral care (13 data points extracted from six of the 16 articles.)						
	Does poor oral health or oral care routines increase the risk of …					
	Pulmonary		Survival		Other	
conclusive	4		1		2	
suggestive	1		0		1	
unclear	1		2		1	
not supportive	0		0		0	
Overall evidence	Strong		Insufficient		Strong	
Lifestyle and Activities of Daily Living (ADLs) (Three data points extracted from three of the 16 articles.)						
	Does level of mobility or lack of physical conditioning or smoking status alter risk of …					
	Pulmonary		Survival		Other	
conclusive	1		0		0	
suggestive	0		0		1	
unclear	1		0		0	
not supportive	0		0		0	
Overall evidence	Insufficient					
Unintended risk factors (Three data points extracted from three of the 16 articles.)						
	Does presence of tubes alter risk of …					
	Pulmonary		Survival		Other	
conclusive	2		0		0	
suggestive	1		0		0	
unclear	0		0		0	
not supportive	0		0		0	
Overall evidence	Strong		Insufficient			

Only questions with data are included in the table. For a complete list of questions and to note questions not addressed in this review, see Table 1. Note that there were no data points extracted for “S” and, therefore, it is not included in this table.

#### 3.2.1 Bolus variables

This investigation explored three questions related to bolus variables: (1) does aspiration of thick or dense material increase risk of an adverse event, (2) does frequent or large amounts of aspiration increase risk of an adverse event, and (3) does aspiration of acidic content increase risk of an adverse event? Twelve datapoints were extracted from the literature and addressed the first two questions. The rapid review did not generate sufficient evidence to determine a relationship between aspiration of thick or dense materials and either pulmonary consequences or mortality.

The rapid review provided strong evidence to support a relationship between aspiration of thick or dense materials and the incidence of other medical complications, such as number of febrile days, dehydration, and urinary tract infections. There is moderate evidence that frequent or large amounts of aspiration increase the likelihood of an adverse event, but there were an insufficient number of data points within any one specific outcome measure.

#### 3.2.2 Oral health and oral care

We explored if poor oral health or oral care increased the risk of an adverse event in those with prandial aspiration. Thirteen datapoints were extracted from the literature and revealed strong evidence between the status of the oral cavity and the risk of pulmonary or other general medical complications in individuals who aspirate. The data are insufficient to confirm a linkage between oral health or oral care and survival.

#### 3.2.3 Lifestyle and activities of daily living

We explored the relationship between the level of physical activity, mobility, and lifestyle choices such as smoking, and the risk of an adverse event in individuals who aspirate. Across the 16 studies, three datapoints were extracted from three studies. There is insufficient evidence in this dataset to determine the strength of the evidence.

#### 3.2.4 Unintended risk factors

We evaluated if the data supports a relationship between the presence of tubes or ventilator and the risk of an adverse event. Across the 16 studies, three datapoints were extracted from three studies to address this question. There is strong evidence regarding the increased risk of a pulmonary consequence when tubes are present in the oropharynx in individuals who aspirate. However, the data were insufficient to determine the relationship between the presence of tubes and the risk of general medical complications or mortality.

#### 3.2.5 System status and general health

There were no datapoints extracted in this rapid review to address this category of risk factors.

## 4 Discussion

This project's aim was to identify the level of support in the literature for the BOLUS framework and dysphagia-related adverse events. This was accomplished using a rapid review. Although this investigation provides an initial assessment of the BOLUS framework's utility, it is worth noting that the rapid review did not identify key articles known to the investigators, which could have enriched this analysis. The inability of this rapid review to identify these articles diminishes the reliability of the conclusions. In fact, in comparison to our previous tutorial on this topic (2), which cited over 100 references to provide support for the BOLUS framework's development, the rapid review process identified only a small subset of those articles. Given the limited number of studies obtained through this rapid review, the conclusion must be viewed in combination with the literature at large.

### 4.1 Bolus variables

The early observations from Langmore et al. (1), that aspiration of thicker consistencies increased the odds of an adverse event, such as aspiration pneumonia, are supported by this investigation. Of the 16 studies identified in the rapid review, six investigated the impact of bolus variables. There was a strong level of evidence to support that aspiration of thick and dense materials increased the likelihood of general medical complications. However, there was insufficient evidence to address the relationship between aspiration of viscous material and pulmonary consequences or survival. A prospective clinical trial by Nativ-Zeltzer et al. (9) evaluated this question using an animal model where they exposed rabbits to controlled amounts of thick liquids or water. In general, thick materials in the lungs reduced the survival rate by about 80%. Further, rabbits with exposure to any thickened material suffered more pulmonary damage than those that were aspirated with water only. Although not identified in the rapid review, these findings were also noted in rats, whereby aspiration of thickened liquid as opposed to water, intensified pulmonary damage and mortality (10). As the primary evidence is animal based, and the direct application of animal lungs and their pulmonary host defenses to human physiology is guarded, these conclusive findings were downgraded to a suggestive level of support. Despite these compelling animal studies, the collective datapoints obtained in the rapid review were insufficient to provide needed support for a stronger level of evidence.

The rapid review showed a moderate level of evidence between the frequency and amount of prandial aspiration and the risk of an adverse event. Since there were only three datapoints on which to evaluate this question, outcome measures were clustered and specific outcomes could not be evaluated. Looking beyond the studies identified in the rapid review, Feinberg and colleagues (11) showed a relationship between amount of aspiration and mortality. This risk was even greater for individuals with frequent and large amounts of aspiration who were receiving non-oral feedings. The incidence of pneumonia in the minor vs. major aspirators reported in the Feinberg et al. study was not significant. However, there was a trend towards a higher incidence of aspiration pneumonia in those with greater aspiration.

The rapid review did not identify any studies that addressed risk associated with pH of aspiration. However, a previous publication by Palmer and Padilla (2) identified literature indicating that aspirated materials are not equitable in chemical composition and host response (12, 13), and that aspiration of large volumes and acid laden contents carries an increased likelihood of negative consequences (14–16). In this rapid review, we eliminated studies that addressed the impact of extra-esophageal reflux. Yet, the literature is replete with studies that link respiratory complication and the presence of GERD [e.g., (17, 18)]. Perhaps data from the impact of GERD on complications can be used to extrapolate that aspiration of acidic content is linked to pulmonary sequalae.

### 4.2 Oral health and oral care

When assessing impact of poor oral health and oral care on the risk of pulmonary complication in individuals who aspirate, the rapid review identified six studies, four of which had conclusive support for the risk of this adverse event. In short, for individuals with suboptimal oral health or oral hygiene, there is an increased likelihood of an adverse pulmonary event (19–23) or other general medical complications (19, 20, 23). In fact, there is a clear reduction of periodontal pathogens (24–26) and risk of aspiration pneumonia when oral care is provided (27). Given the conclusive data in the rapid review, as well as the additional available literature, adequate and consistent oral care that includes brushing and rinsing the oral cavity, should remain a high priority for individuals with prandial aspiration.

The rapid review did not yield sufficient datapoints to draw definitive conclusions regarding the relationship between oral health or oral care and survival. Three datapoints were identified that addressed this relationship. Gosney and colleagues (20) reported three deaths during their study, which involved selective decontamination and monitored bacteria, episodes of pneumonia, and death. Interestingly, all three deaths occurred in the placebo group, while none were reported in the treatment group that received decontamination. Although selective decontamination showed a decrease in gram-negative bacteria colonization and the documented number of cases of aspiration pneumonia, it did not impact mortality. In this rapid review, only one of the three datapoints provided conclusive support for a relationship between oral care and survival (23).

### 4.3 Lifestyle and activities of daily living

None of the identified studies addressed dependence for oral care and feeding. Further, there were insufficient datapoints to thoroughly examine the relationship between the risk of an adverse event and factors such as mobility, physical activity, and lifestyle choices. However, Karaginnis et al. (28) reported that of the six patients who had sequelae from aspiration *all* had limited to no mobility. These findings parallel existing literature, not identified in this rapid review, which suggests that mobile individuals are less prone to aspiration compared to non-ambulatory individuals and have a lower likelihood of pneumonia if not bed-ridden (29, 30). The underlying hypothesis is that mobility aids in pulmonary clearance. In stark comparison, frail individuals with swallowing impairment, who are presumed to be less mobile, have a higher level of mortality (4).

Lifestyle choices such as smoking, have been associated with an increased risk of post-operative pneumonia in individuals following cardiac surgery (19), corroborating Langmore et al.'s findings on the impact of smoking on risk of an adverse event (1, 30). Indeed, current active smoking diminishes the effectiveness of the mucociliary escalator (31), thereby rendering the host's response efficacy. Overall, while there is literature to support the relationship between physical activity and lifestyle choices and the risk of an adverse event, the rapid review did not support nor negate this relationship.

### 4.4 Unintended risk factors

No studies addressed the impact of medication on saliva (i.e., production amount or viscosity), alertness or cognitive function. Based on a limited dataset of three observations, strong evidence emerges to support a link between iatrogenic risk factors and the likelihood of encountering an adverse event from aspiration. Research indicates that individuals with shorter duration of feeding tube usage, or those that employ intermittent (i.e., placed for meals only) feeding tube placement experienced more favorable outcomes (32, 33). Ickenstien et al. (32) reported a positive relationship between shorter duration of feeding tube placement and improved survival rates. The consequences associated with the presence of a tube is likely linked to the microbial growth that adheres to that tube (34).

Although the rapid review failed to uncover studies investigating the impact of a tracheotomy tube on adverse events in those with dysphagia, a retrospective cohort study by Nativ-Zeltzer et al. (3), supports the conclusion that the presence of a tracheotomy tube in an individual with swallowing impairment increases the likelihood of aspiration pneumonia. Despite the prevailing evidence suggesting an increase in risk associated with the presence of tubes, Langmore et al. (30) found no relationship in nursing home residents, which stands in contrast to our strong evidence.

### 4.5 System status and general health

There were no studies identified in this rapid review for system status. However, given current literature, when individuals with dysphagia have respiratory disease, GI disease, or when these co-occur, the odds of a negative outcome rise (1, 4, 30). The lungs are a vital component of the host defense mechanism. A lung without pathology is more resilient to injury and invasion as compared to a pulmonary system with chronic disease (35). This reduced host response is exacerbated when the individual is also frail and has suboptimal nutrition status (36, 37). Further, when discussing reactive airway protective mechanisms, we must not overlook the vital role that cough plays (38, 39). The strength of a cough as measured by peak flow can help identify those at increased odds for mortality (40).

### 4.6 Summary of support for BOLUS framework

In summary, additional evidence to support the BOLUS framework was obtained. With strong to moderate evidence for an increased risk of a pulmonary consequence or other general medical complication when there is aspiration of thick material, or in the presence of poor oral care or a tube. There was insufficient evidence to address the risk of mortality related to aspiration. In this rapid review, there was insufficient evidence regarding the impact of system status. Therefore, a more thorough review such as a systemic review should be employed.

### 4.7 Limitations

This study employed a rapid review methodology to efficiently assess current data. Consequently, studies previously discussed in a literature review by Palmer and Padilla (2) were not retrieved using the search criteria employed in this current rapid review. The limited number of identified studies in this rapid review diminished the comprehensiveness of this investigation. Integrating pivotal articles from the previous publication by Palmer and Padilla (2) would enhance the robustness and depth of the conclusions drawn from this rapid review.
