# Supplementary material for: Linking the impact of aspiration to host variables using the BOLUS framework: support from a rapid review
Source: Front Rehabil Sci. 2024 Jul 12;5:1412635. doi: 10.3389/fresc.2024.1412635 (PMC11273940; doi:10.3389/fresc.2024.1412635)

**11 SUPPLEMTAL MATERIALS**

**11.1 Figure 3.** Summary of results from the rapid review using a preferred reporting items for systematic review and meta-analysis (PRISMA) format.

**
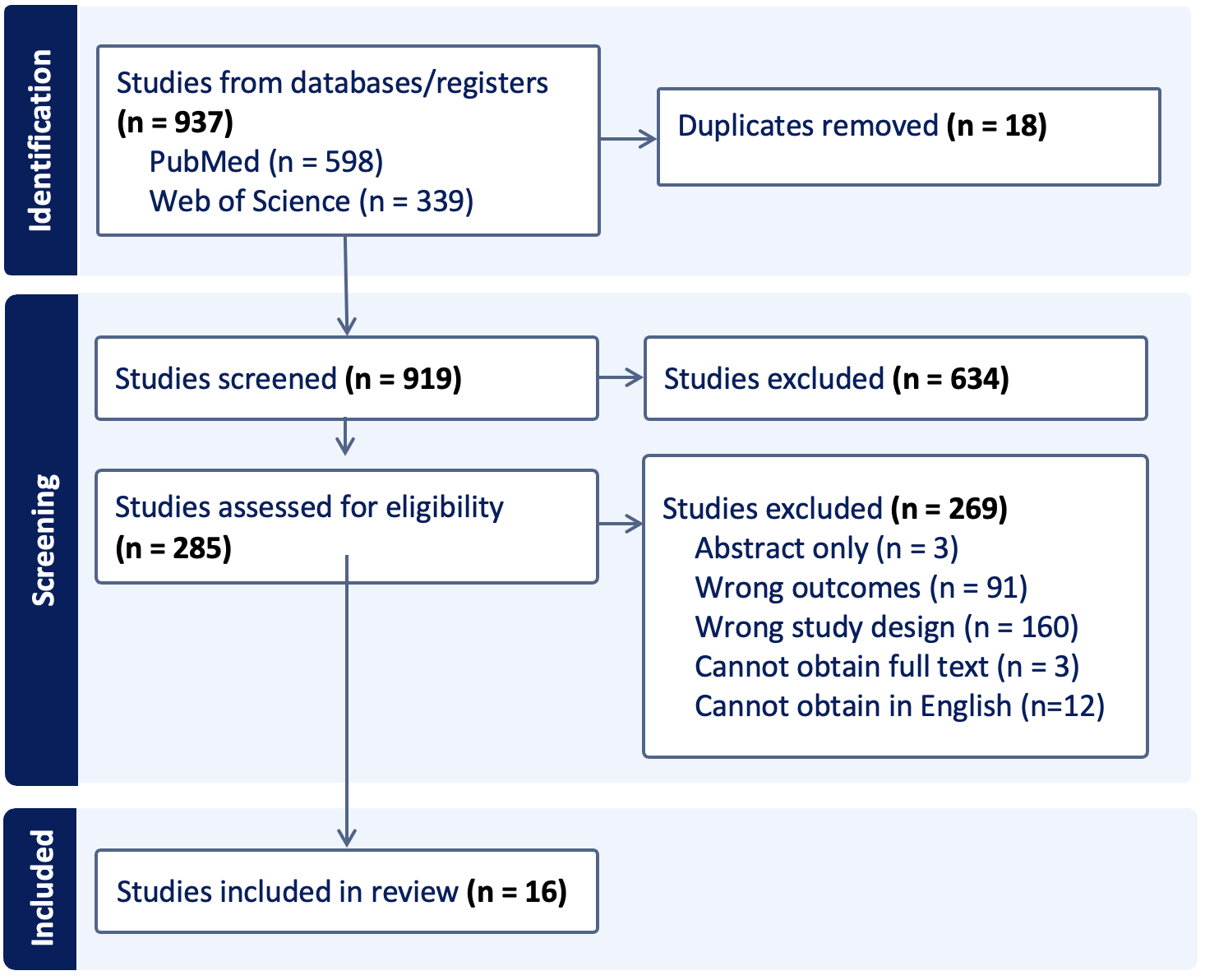
**

**11.2 Appendix A: Video Resources**

The following YouTube videos provide an overview of the BOLUS framework.

- Part 1: Aspiration and the Pulmonary Biome. Here we review lung physiology and the impact of aspiration
  - <https://www.youtube.com/watch?v=JVq0iQJrbp4&t=35s>
- Part 2: A review of current evidence to guide clinical decision making
  - <https://www.youtube.com/watch?v=BzisjH7xqj8&t=14s>
- Part 3: Proposal of the BOLUS framework and application to two case studies.
  - <https://www.youtube.com/watch?v=C0lUHjSJ1uw&t=64s>

**11.3 Appendix B**

Comparison of the citations identified in the rapid review to those used to support the development of the framework in Palmer and Padilla (2022). Highlighted citations indicate that a given citation is present in both the rapid review and the tutorial article. A strikethrough indicates that it was cited in Palmer and Padilla but eliminated in the rapid review process. All other citations occurred in either the tutorial or the rapid review.

|  | Questions from Palmer & Padilla (2022) | Associated citations from Palmer & Padilla (2022) | Associated citations from the Rapid Review |
| --- | --- | --- | --- |
| B | Is my patient aspirating thickened liquids, solids, or highly acidic materials?  Is my patient frequently aspirating in large volumes? | - Cichero & Lam, 2014 - ~~Kaneoka et al., 2017~~ - Langmore et al., 1998 - Mandell & Niederman, 2019 - ~~Marik, 2001~~ - Miles et al., 2018 - Murray et al., 2016 - Nativ-Zeltzer et al., 2018 - Nativ-Zeltzer et al., 2020 - Neill & Dean, 2019 - Raghavendran et al., 2011 - Robbins et al., 2008 - Shim et al., 2013 - Son et al., 2017 | - Ickenstein et al., 2005 - Karagiannis et al., 2011 - Murray et al., 2016 - Masuda et al., 2022 - Nativ-Zeltzer et al., 2018 - Robbins et al., 2008 |
| O | Does my patient have evidence of oral cavity neglect?  Does my patient have poor oral care routines?  Does my patient have reduced oral saliva? | - Dewhirst et al., 2010 - Dyck et al., 2012 - El-Solh et al., 2004 - ~~Johnson & Hirsch, 2003~~ - Kageyama et al., 2017 - Kageyama et al., 2018 - Langmore et al., 2002 - Langmore et al., 1998 - Sarin et al., 2008 - Seedat & Penn, 2016 - Sudhakara et al., 2018 - Brosky, 2007 - Dawes & Wong, 2019 - Liebsch et al., 2019 - Mosca & Chen, 2017 - Thul et al., 2018 | - Adachi et al., 2007 - Carrel et al., 2001 - Gosney et al., 2006 - Hollaar et al., 2017 - Kucukcoskun et al., 2013 - Sorensen et al., 2013 |
| L | Is my patient dependent on others for oral care?  Is my patient dependent on others for feeding?  Is my patient active and mobile? | - Gosselink et al., 2008 - Hathaway et al., 2014 - Lai et al., 2017 - Langmore et al., 1998 - Langmore et al., 2002 - McNally et al., 2019 - Quinn et al., 2014 - Tay et al., 2014 - Wang et al., 2018 - Wright et al., 2008 | - Karaginnis et al., 2011 - Fujimaki et al., 2017 |
| U | Does my patient have tubes that harbor bacteria?  Is my patient receiving mechanical ventilation?  Is my patient on medications that alter cognition or attention?  Is my patient on medications that alter saliva (viscosity or amount)? | - Blumenstein et al., 2014 - Berdal et al., 2007 - Brodsky et al., 2020 - Gomes et al., 2003; - Herzig et al., 2009 - Katzberg & Benatar, 2011 - Kurien et al., 2017; - Langmore et al., 1998 - Leibovitz et al., 2003 - Li Bassi et al., 2008 - McClave & Chang, 2003 - Pancorbo-Hidalgo et al., 2001 - Park et al., 2019 - Pisegna & Langmore, 2018 - Slutsky & Ranieri, 2013 - Takayama et al., 2017 - Teno et al., 2012 - Torres et al., 1992 | - Freytag et al., 2003 - Ickenstein et al., 2005 - Juan et al., 2020 |
| S | Is my patient in poor general health?  Is my patient frail or deconditioned?  Does my patient have reduced cognitive function that impacts swallow safety?  Does my patient have reduced respiratory function (pulmonary/respiratory disease)?  Does my patient have a weak cough?  Does my patient have gastroesophageal reflux disease or gastrointestinal disease?  Does my patient have compromised immune function? | - Abdulkhaleq et al., 2018 - Abe et al., 2011 - Agostino et al. (2020) - Balou et al. (2019) - Bock et al., 2017 - Burtin et al., 2009 - Bustamante-Marin & Ostrowski, 2017 - Cesari et al., 2016 - Crisafulli et al., 2007 - DiBardino & Wunderink, 2015 - Dickson, 2016 - Easterling & Robbins, 2008 - El-Solh et al., 2011 - Eurich et al., 2010 - Gaude, 2009 - Gendrel & Bohuon, 2000 - Hathaway et al., 2014 - Herzig et al., 2009 - Humbert et al., 2010 - Hutcheson et al., 2018 - Jo et al., 2017 - Kayambu et al., 2013 - Keller, 2019 - Kim & Sapienza, 2005 - Knowles & Boucher, 2002 - Laheij et al., 2005 - Langmore et al., 1998 - Langmore et al., 2007 - Langmore et al., 2002 - Legriel et al., 2019 - Leopold et al., 2009 - Li et al., 2007 - Mayadas et al., 2014 - Mills & Ashford, 2008 - Morris et al., 2018 - Ovechkin et al., 2016 - Pitts et al., 2009 - Rösler et al., 2015 - Shamliyan et al., 2013 - Sprouls et al., 2014 - Vintimilla et al., 2018 - Walston et al., 2006 - Weiskopf et al., 2009 - Xue, 2011 - Yao et al., 2011 |  |


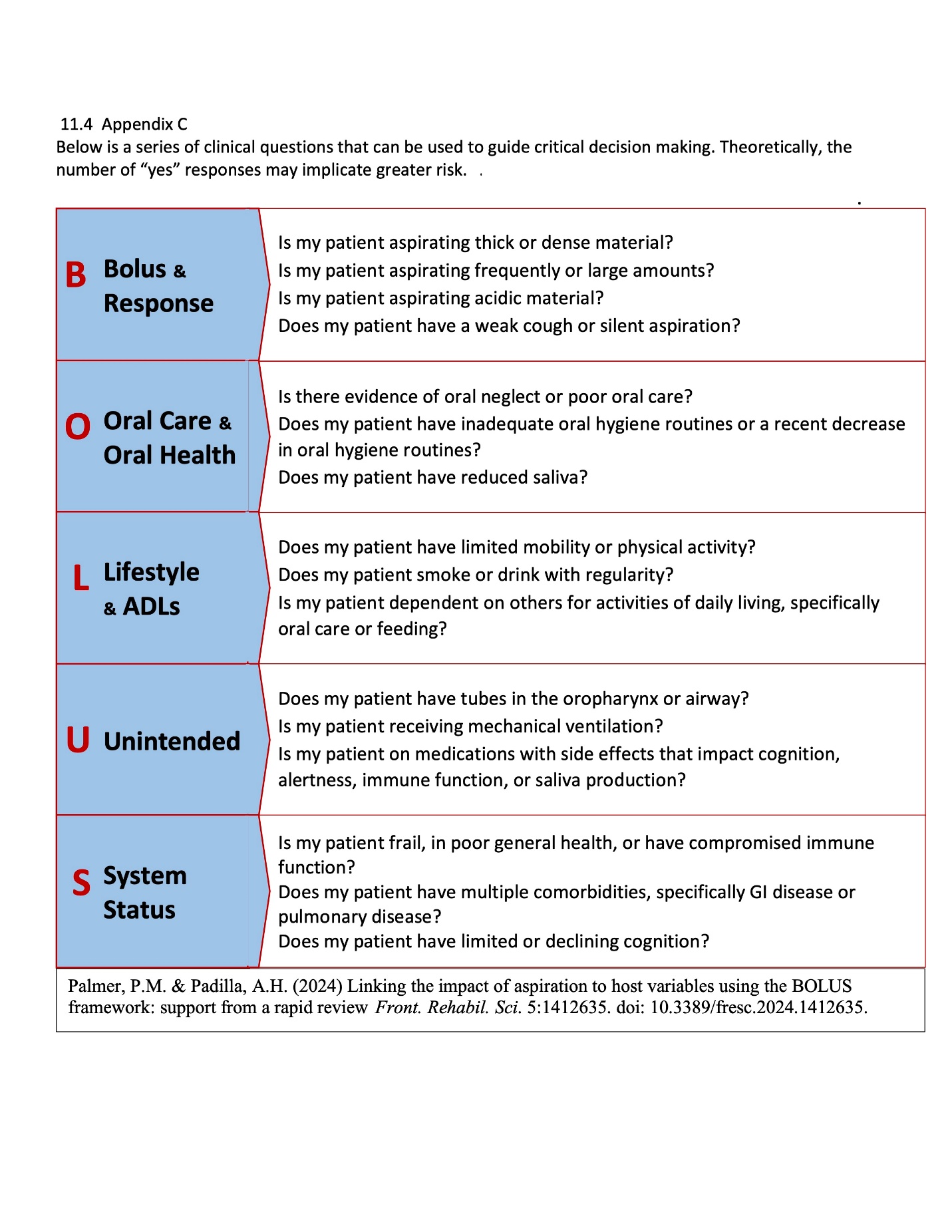

Supplement: Supplementary file 1 [file Datasheet1.docx]
